# Machine learning prediction of multiple distinct high-affinity chemotypes for α-synuclein fibrils

**DOI:** 10.1039/d5cc06228d

**Published:** 2026-01-13

**Authors:** Xinning Li, Ryann M. Perez, Zhude Tu, Robert H. Mach, Sam Giannakoulias, E. James Petersson

**Affiliations:** a Department of Chemistry, School of Arts and Sciences, University of Pennsylvania 231 South 34th Street Philadelphia PA 19104 USA ejpetersson@sas.upenn.edu; b Department of Radiology, Washington University School of Medicine St Louis MO 63110 USA; c Department of Radiology, Perelman School of Medicine, University of Pennsylvania 3400 Spruce Street Philadelphia PA 19104 USA; d Division for Advanced Computation, Sentauri Inc. Glenwood MD 21738 USA; e Department of Biochemistry and Biophysics, Perelman School of Medicine, University of Pennsylvania 421 Curie Boulevard Philadelphia PA 19104 USA

## Abstract

To identify new ligands for positron emission tomography imaging of α-synuclein aggregates, we developed a machine learning model trained on <300 binding measurements. We used scaffold-guided curation to select a 30 compound prospective set from a 140-million-member library. Experimental validation yielded five high-affinity binders, showing robust generalization for ligand discovery.

## Abstract 2

Machine learning model predicts α-synuclein ligands.

## Graphical abstract

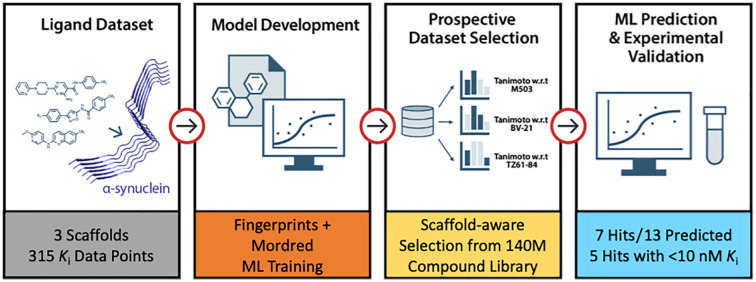

The synucleinopathies are a set of neurodegenerative diseases characterized by aggregation of the protein α-synuclein (αS) that include Parkinson's disease (PD), multiple system atrophy (MSA), and Lewy body dementia (LBD).^1^ PD is the second most common neurodegenerative disorder, behind Alzheimer's disease (AD); collectively these diseases affect 15% of the global population, a figure projected to double by 2050 with increasing human lifespan.^2^ Staining of αS aggregates in post-mortem brain tissue slices has been crucial to understanding the pathology of PD, MSA, and LBD, but there is a need for tools to image the progression of synucleinopathies *in vivo*, which would provide biomarkers for guiding the development of αS-targeting therapies for PD and MSA.

Small molecules that specifically bind to αS fibrils could serve as positron emission tomography (PET) imaging probes to study disease progression or act as tools for early clinical diagnosis of synucleinopathies.^3^ Indeed, such PET probes have proven invaluable for studying AD progression and evaluating the efficacy of therapeutics.^4^ PET ligand development in our laboratories has used rational design principles for structure–activity relationship (SAR) optimization as well as computational methods encompassing both similarity searches for analogs of existing hits and docking-based ultra-high throughput screens for new scaffolds.^5–9^ These studies have provided three major classes of ligands, typified by the compounds BV-21, TZ61-84, and M503 (Fig. 1). All three of these molecules have shown affinities of <5 nM for *in vitro* αS fibrils in direct binding assays using radioisotope labeled analogs.^6,7,9^ This has translated to high affinities in human PD and/or MSA tissue homogenates, and selective binding in autoradiography experiments that have allowed M503 and a structural analog, HY-2-15 (SI, Fig. S1), to move forward to initial human validation.^10,11^ In the course of developing these ligands, we have carried out thousands of ligand binding experiments, and this database now allows us to develop machine learning (ML) models to guide the identification of new ligands from among the three structural classes or deriving from hybrid or completely new scaffolds. In particular, we will focus on ML models for classifying displacement of the tritiated BF-2846 radioligand (Fig. 1), for which >300 inhibition constant (*K*_i_) values are available to be used in model training.

**Fig. 1:**
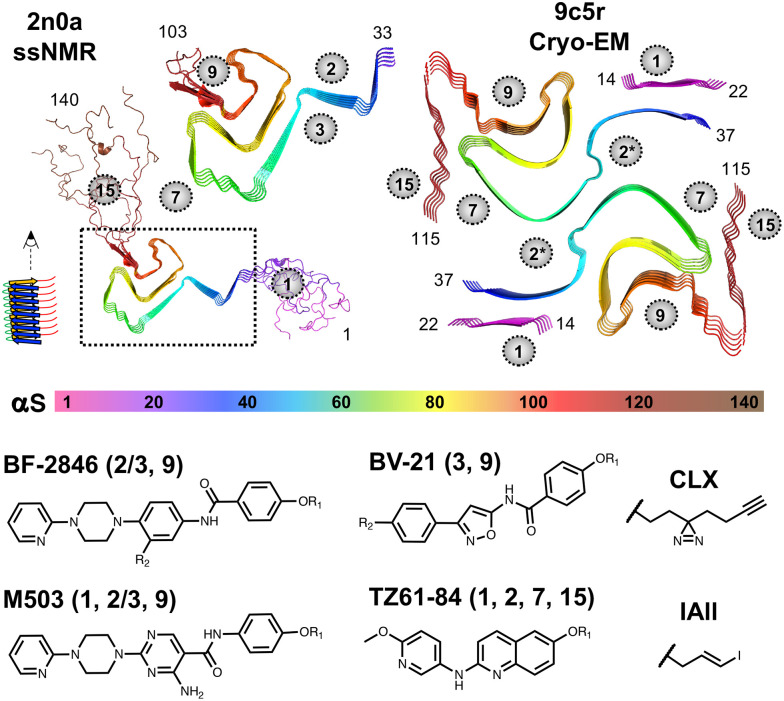
αS Fibril and ligand structures. Top: *In vitro* ssNMR or cryo-EM fibril structures rendered from noted PDB ID coordinates and colored according to the rainbow scheme below the structures.^12,16^ Binding sites identified in computational analysis of PDB ID 2n0a are noted with grey circles.^13^ Bottom: Candidate PET ligand structures and crosslinkable analogs used in XL-MS. BF-2846: R_1_ = C^3^H_3_, R_2_ = H used for radioligand binding, R_1_ = CH_3_, R_2_ = N_3_ or R_1_ = CLX, R_2_ = H used for XL-MS. BV-21: R_1_ = CH_3_, R_2_ = ^125^I used for radioligand binding, R_1_ = CLX, R_2_ = H used for XL-MS. M503: R_1_ = C^3^H_3_ used for radioligand binding, R_1_ = CLX used for XL-MS. TZ61-84: R_1_ = ^125^IAll used for radioligand binding, R_1_ = CLX used for XL-MS. For each ligand, the binding sites identified in XL-MS experiments are noted in parentheses.

Interpreting αS fibril binding experiments presents two challenges that are not found in typical drug discovery investigations: polymorphism of the fibril structure and multiple high affinity binding sites for each ligand. Our development of PET ligands for PD, LBD, and MSA began with the identification of 10–15 potential ligand binding pockets observed in the original solid-state NMR (ssNMR) structure of αS fibrils (PDB ID: 2n0a, shown in Fig. 1).^12,13^ Site 2 is located near residues Y_39_-E_46_ in the ssNMR structure, and site 9 is located near residues G_86_-K_96_. These sites presented the deepest binding pockets that were surface accessible in the ordered regions of the fibril. More recent cryo-EM structural data show that *in vitro* αS fibrils commonly exist as multi-protofilament structures and that this can result in a rearrangement of site 2 and site 3 (backbone rotation that places Y_39_ and H_50_ on the same face of the protofilament) to form a site that we refer to as site 2* (Fig. 1).^14^ Our own recent studies on the binding of site 2* ligands indicate that the conditions used for fibril preparation in our studies generate fibrils with a two-standed morphology (PDB ID: 9o4b),^15^ and a more ordered N-terminus, similar to that recently reported by Jiang *et al.* (PDB ID: 9c5r).^16^ The more ordered N-terminus presents site 1 (G_14_-T_22_), as a potential ligand-binding location, as well as a portion of the C-terminus that we refer to as site 15 (D_115_-E_123_). We interpret our binding data with *in vitro* fibrils primarily in terms of the 9c5r structure, since this recapitulates features of other *in vitro* αS fibril folds, and is consistent with photo-crosslinking mass spectrometry (XL-MS) data used in identifying ligand binding sites.

XL-MS data for photo-crosslinkable (CLX) derivatives of our ligands have been mapped onto the *in vitro* ssNMR and cryo-EM structures to allow us to understand where binding of BV-21, M503, and TZ61-84 overlap with binding of BF-2846 (Fig. 1).^5,9,17^BV-21 XL-MS data identifying site 3 are consistent with our cryo-EM structures of the binding of related compound Ex-6.^15^ The primary areas of overlap are at site 2*(sites 2/3) for all ligands and site 9 for all ligands but TZ61-84. These data provide a structural understanding of the basis for [^3^H]BF-2846 displacement by the other ligands and also demonstrate the potential for some binding events that may not be observed in the radioligand assays (*e.g.*, TZ61-84 class compound binding at site 1 or site 15). While the relative affinities for the sites may vary, the XL-MS data support the use of [^3^H]BF-2846*K*_i_ measurements in developing classifiers for αS fibril binding across three types of ligand structures.

To develop our ML model for predicting αS fibril binding affinity in [^3^H]BF-2846 displacement, we curated 315 experiments in which full 10-point binding curves were collected, resulting in either a fitted *K*_i_ value or an assessment of “no binding” if a curve could not be fit with *K*_i_ < 1 µM. For these 315 measurements, there is reasonable class balance, with 138 BF-2846/M503 class compounds, 121 BV-21 class compounds, and 56 TZ61-84 class compounds. About 1/3 of the compounds (99) have low similarity to any of the class parents, showing reasonable diversity in the data set. A deeper analysis of similarity is given in SI, Fig. S2 and S5. This class balance and diversity is representative of a typical drug screening campaign, where there will often be a skew toward known hits.

Building on our experimental data, we trained an ML model to identify potential binders for αS fibrils from the full-scale Mcule library, comprising approximately 140 million compounds.^18^ Using 315 full binding measurements, our model achieved consistent performance on cross-validation and the held-out test set. We then applied the model to a scaffold-diverse 30 compound prospective library and identified 13 predicted binders. Experimental validation confirmed seven true binders, including five compounds with <10 nM *K*_i_ spanning multiple chemotypes. These results demonstrate that the model generalizes beyond the training distribution and provides practical value for identifying potent αS PET probe candidates.

Binder labels were assigned using a 25 nM *K*_i_ threshold: compounds with *K*_i_ <25 nM were classified as binders, whereas those with *K*_i_ >25 nM were designated as non-binders. This cutoff was chosen for two reasons. First, 25 nM represents a meaningful potency level that helps distinguish promising, high-affinity ligands. Second, this cutoff provides a balanced distribution of binder and non-binder classes in the dataset. Lower thresholds would lead to an imbalanced set dominated by non-binders, whereas higher thresholds would incorporate weaker ligands that are less relevant for our application. To ensure representative training and test sets, we applied stratified splitting to preserve the class distribution across datasets (SI, Table S1). Given the limited size of the training set (271 data points), we selected algorithms with inherently higher bias and lower variance to reduce overfitting and achieve robust generalization.^19^ Specifically, we trained logistic regression, k-nearest neighbors, and decision tree classifier models. Molecules were featurized for classical ML using a combined feature-set of Morgan fingerprints and Mordred chemical descriptors. Together, these features capture both bulk and substructural information from molecules to be used in ML.^20,21^ To maximize performance, we tuned hyperparameters and performed feature selection *via* five-fold cross-validation, optimizing for the macro *F*1 score. Macro *F*1 was chosen as the primary optimization metric because the dataset is highly imbalanced, and accurate prediction of the minority (binder) class is of particular importance. In parallel, to provide an intuitive baseline, we created a simple similarity-based classifier: Morgan fingerprints (radius 3, 1024 bits) were computed for all compounds, and each molecule was assigned a binder label if its maximum Tanimoto similarity to any training-set binder exceeded a threshold *τ*. We selected *τ* = 0.5 as it reflects meaningful structural similarity, while higher values would capture only near-identical compounds. This yielded a baseline macro *F*1 score of 0.56. Classification reports for the best-performing models selected by cross-validation and for the similarity-based baseline classifier are provided in the (SI, Tables S2–S5).

The logistic regression model achieved the highest performance and was therefore selected as the final model for subsequent binder predictions (SI, Table S6 and S7; Table 1). The optimal hyperparameters are listed in SI, Table S8, and the selected features are available on our GitHub repository. To aid interpretation, the top 40 logistic regression feature coefficients are shown in SI, Fig. S4. The final model achieved a weighted *F*1 score of 0.79 and a macro *F*1 score of 0.72 during cross-validation (SI, Table S2). When evaluated on the test set, the model maintained consistent performance, with identical weighted and macro *F*1 scores of 0.79 and 0.72, respectively (Table 1). Moreover, relative to the best cross-validation results, the model exhibited improvements in weighted precision, macro precision, and macro recall (Δweighted-precision = +0.02; Δmacro-precision = +0.02; Δmacro-recall = +0.02), while maintaining stable weighted recall across datasets. These results collectively indicate that the model not only fits the training data effectively, but also preserves predictive reliability when exposed to unseen samples. The comparable performance between cross-validation and test evaluations suggests minimal overfitting and robust model generalization. Importantly, achieving this stability and accuracy with a small and somewhat imbalanced test set that includes only 9 binders among 44 total samples highlights the model's capacity to capture meaningful discriminative patterns for αS fibril binding within such data constraints. This level of performance underscores the potential applicability of the model in scenarios where experimental data are limited, but accurate binder prediction remains critical.

**Table 1:** Classification report on test set

	Precision	Recall	*F*1
Non-binder	0.96	0.74	0.83
Binder	0.47	0.89	0.62
Accuracy			0.77
Macro	0.72	0.81	0.72
Weighted	0.86	0.77	0.79

To externally validate the model, we developed a scaffold-aware workflow to curate a prospective dataset from the full Mcule library for model inference. Guided by prior SAR analysis highlighting the three reference chemotypes (BV-21, M503, and TZ61-84), we assigned each library molecule to its nearest reference scaffold using Tanimoto similarity. Candidates were classified according to their closest chemotype, which allows us to assess the extent of scaffold coverage within the dataset (Fig. 2, grey). Overlaying Tanimoto scores of the compounds in the training and test sets (Fig. 2, red) shows that we have a range of chemotypes, not just close analogs of BV-21, M503, and TZ61-84. For prospective compound set selection, we analyzed the Mcule database with the same Tanimoto metrics. We then deliberately selected a small number of high Tanimoto compounds (including some non-Mcule molecules for TZ61-84-type representation), but also sampled low Tanimoto scores (<0.2 to BV-21, M503, and TZ61-84 for 16 compounds) to expand chemical space and assess model generalization. This scaffold-aware, yet diversity-oriented selection method for our 30 compound prospective set (Fig. 2, blue) avoids collapsing onto a single chemotype while rigorously evaluating the model's capability to generalize beyond its training domain.

**Fig. 2:**
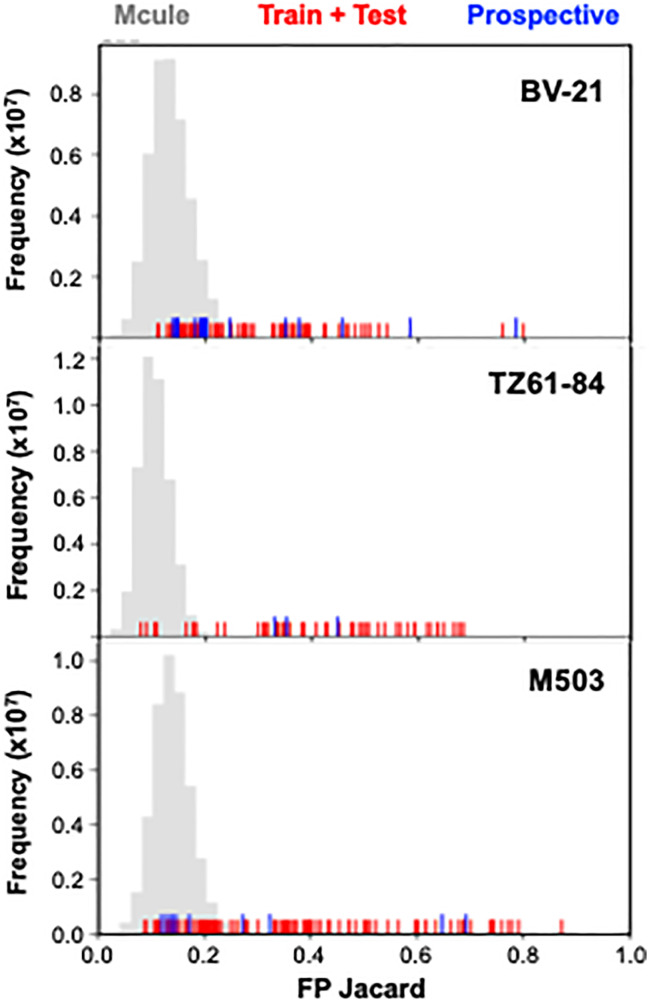
Scaffold similarity distributions for training, test, and prospective datasets. Grey histograms represent distributions of Tanimoto similarity values between Mcule library compounds and the three reference chemotypes (BV-21, M503, and TZ61-84). Red ticks mark similarity values for training and test compounds, and blue ticks mark those for the prospective compounds.

Using our ML model, we identified 13 potential binders (Fig. 3). To validate the predictions, we performed a [^3^H]BF-2846 assay to determine *K*_i_ values. Based on these experimental labels, we obtained a weighted *F*1 score of 0.74 and a macro *F*1 score of 0.71 (SI, Table S11). Despite a modest decrease in performance compared to the test set, the results remain noteworthy given that the model was trained on only 271 data points, yet generalized effectively to a diverse dataset. Importantly, although the final model was selected based on its performance on a small test set, that performance closely matched the cross-validation results and was again reproduced in the prospective evaluation, indicating that the observed accuracy reflects genuine model generalization.

**Fig. 3:**
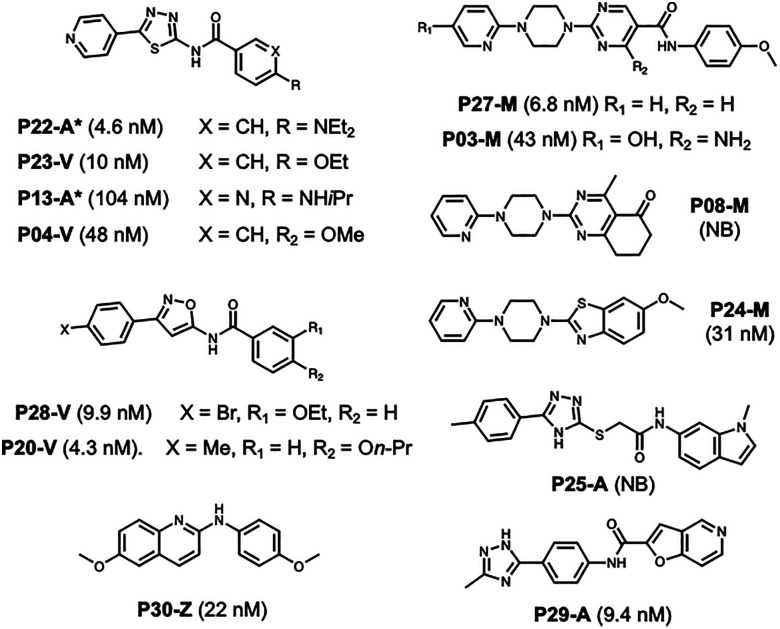
Predicted hits from prospective compound set. Compound names indicate structural class (V = BV-21, M = M503/BF-2846, Z = TZ61-84, A = Alternative/Hybrid). Classes assigned based on Tanimoto scores, where A indicates that scores relative to BV-21, M503, and TZ61-84 were all <0.2. *Despite apparent similarity to BV-21, these compounds have low Tanimoto scores due to the combination of thiadiazole and exocyclic amine. Experimental *K*_i_ values g_i_ven in parentheses. The full list of compounds in the prospective set with associated computational and experimental data is given in SI Table S12.

Importantly, the model demonstrated strong capability in filtering non-binders, achieving an *F*1 score of 0.79 for non-binder prediction. This high performance allows for efficient prioritization of compounds by reducing the experimental burden of testing inactive molecules. Moreover, the model successfully identified seven strong binders, five of which exhibited *K*_i_ values below 10 nM, underscoring its potential to guide hit discovery (data for all 30 compounds in SI, Table S12). The 13 predicted hit compounds are shown in Fig. 3. Notably, the true positives include at least one compound from each of the structural classes as well as alternative scaffold compounds. Among the false positives are three compounds with *K*_i_ values <50 nM, still potent compounds, although above our 25 nM cut-off. While the alternative scaffolds share elements of existing scaffolds, they provide new areas of chemical space to explore.

In summary, we report a data-efficient ML framework to accelerate the identification of high-affinity αS fibril PET ligand candidates. Despite being trained on only 271 data points, the logistic regression model integrated feature selection and exhibited generalization across chemical space, enabling efficient prioritization of active scaffolds. Prospective validation against a curated Mcule dataset yielded five compounds with sub-10 nM *K*_i_s, including BV-21-type hits with thiadiazoles, which human analysis predicted to be non-binders.^22^ Our scaffold-aware design ensures broad coverage of both known and novel αS-binding chemotypes, providing a generalizable method for leveraging limited biochemical data to guide PET probe design, advancing *in vivo* imaging of synucleinopathies. Although the approach is validated only for this target, it illustrates what can be achieved in ligand discovery when limited high-quality biochemical measurements are combined with an appropriately designed model.

The manuscript was written by X. L. and E. J. P., with input from all authors. X. L., S. G., and E. J. P. designed all experiments and X. L. performed most experiments. R. M. P. curated experimental data.

This research was supported by a grant from the National Institutes of Health to R. H. M. and E. J. P. (NIH U19 NS110456). X. L. thanks Sentauri, Inc. for funding. R. M. P. thanks the NIH for funding through the Chemistry Biology Interface Training Program (T32 GM133398) and an Individual Predoctoral Fellowship (F31 AG090063).

## Conflicts of interest

Sentauri, Inc is a for-profit institution which works as a life science computational consultancy. E. James Petersson acts as a scientific advisory board member of Sentauri.

## Supplementary Material

CC-062-D5CC06228D-s001

CC-062-D5CC06228D-s002

## Data Availability

The data supporting this article have been included as part of the supplementary information (SI) and are available online at https://github.com/ejp-lab/EJPLab_Computational_Projects/tree/master/%CE%B1-SynucleinBinder. Compound synthesis and binding studies for previously unreported compounds in training and test sets are described in an accompanying publication.^22^ Supplementary information: experimental methods, supplemental figures and tables, and associated references. See DOI: https://doi.org/10.1039/d5cc06228d.
