# Supplementary material for: Machine learning prediction of multiple distinct high-affinity chemotypes for α-synuclein fibrils
Source: Chem Commun (Camb). 2026 Jan 13;62(9):2922–6. doi: 10.1039/d5cc06228d (PMC12797025; doi:10.1039/d5cc06228d)
Supplement: CC-062-D5CC06228D-s001 [file CC-062-D5CC06228D-s001.pdf]

Supplementary Information (SI) for ChemComm.
This journal is © The Royal Society of Chemistry 2026

Electronic Supplementary Information

Machine Learning Prediction of Multiple Distinct High-Affinity Chemotypes for α-Synuclein Fibrils

Xinning Li,[a] Ryann M. Perez,[a] Zhude Tu,[b] Robert H. Mach,[c] Sam Giannakoulias,[d] and E. James Petersson*[a,e]

[a] Department of Chemistry, School of Arts and Sciences, University of Pennsylvania, 231 South 34th Street, Philadelphia, PA 19104, USA.

[b] Department of Radiology, Washington University School of Medicine, St Louis, MO, 63110, USA.

[c] Department of Radiology, Perelman School of Medicine, University of Pennsylvania, 3400 Spruce Street, Philadelphia, PA 19104, USA.

[d] Division for Advanced Computation, Sentauri Inc., Glenwood, MD 21738, USA.

[e] Department of Biochemistry and Biophysics, Perelman School of Medicine, University of Pennsylvania, 421 Curie Boulevard, Philadelphia, PA 19104, USA.

*ejpetersson@sas.upenn.edu

Table of Contents:

1. **Software…………………………………………………………………………………… S3**

2. **Radioligand Competition Experiments…………………………………………………… S3**

3. **Data Fitting…………………………………………………………………………………. S4**

4. **Structure of HY-2-15 …………………………………………………………………... S5**

5. **Machine Learning Datasets ……………………………………………………............... S6**

6. **Model Training …………………………………………………………………....... S7**

7. **Feature Analysis ………………………………………………………………...... S12**

8. **Analysis of Training/Test Set Diversity …………………………………………….....S14**

9. **Binder Detection from Mcule library ……………………………………………....... S16**

10. **Reference ……………………………………………………………......……… S20**

Software

The software used in this work is available in the conda environment YAML files at: https://github.com/ejp-lab/EJPLab_Computational_Projects/blob/master/%CE%B1-SynucleinBinder/environment.yml. The environment file defines the computational setup required to reproduce the analyses performed in this study, including all necessary Python packages, dependencies, and version specifications.

Radioligand Competition Experiments

α-synuclein fibrils (50 nM) were mixed with site 9 ligand [^{3}H]BF-2846 (3 nM) and varying concentrations of competitor compounds. Compounds were diluted in 50 mM Tris-HCl (pH 7.4) and mixed with fibrils and radioligand in a total volume of 150 μL. Total binding was measured in the absence of competitor and non-specific binding was determined in reactions containing unlabeled BF-2846 (0.5 μM). In a duplicate set of binding reactions, fibrils were replaced with equal volume of buffer to measure the amount of radioligand binding to the filter paper. Reactions were incubated at 37 °C for 1 hour. After incubation bound and free radioligand were separated by vacuum filtration through Whatman GF/C filters (Brandel) in a 24-sample harvester system (Brandel), followed by washing with buffer containing 10 mM Tris-HCl (pH 7.4) and 150 mM NaCl. Filters containing the bound ligand were mixed with 3 mL of scintillation cocktail (MicroScint-20, PerkinElmer; Waltham, MA, USA) and counted after 12 hours of incubation on a MicroBeta System (PerkinElmer). Counting of all samples was performed in triplicate and mean values computed for radioligand binding analyses.

Data Fitting

To rigorously and consistently fit radioligand binding data and estimate uncertainty, we used a more sophisticated fitting algorithm proposed by Janssen et al. [1]. To initially fit the data, we used the following system of equations:

$$Y = Bottom + (Top - Bottom) / (1 + 10 ** ((log\_conc - logIC50) * HillSlope))$$

$$IC50\_nM = Ki\_nM * (1 + hot\_ligand\_conc / kd\_nm)$$

Where Y is the signal of the radioligand, Bottom is the estimated bottom plateau value, Top is the estimated Top plateau value, log_conc is the logarithm of the competitor concentration, logIC50 is the log of the IC50 value for a given competitor, Ki_nM is the K_i in nanomolar, hot_ligand_conc is the concentration of hot ligand in the assay, and kd_nm is the K_d of the radioligand. Scipy was used to optimize the curve fit over 50000 iterations. After the initial fit, if the curve had datapoints that were beyond 2σ from the initial fit, the outliers were removed, and the curve was refit. For compounds with low uncertainty for various parameters (See Github for more information), these were defined as good. The hill slope of good was imputed on those with poorly defined parameters and the curve fit process was repeated. For curves with yet still undefined Bottoms, determined as having a Bottom estimation with high uncertainty, they were reclassified as non-binding. In total, 12 ligands changed their binding class for the final machine learning dataset.

Structure of HY-2-15

HY-2-15 is a structural analog of M503.

HY-2-15

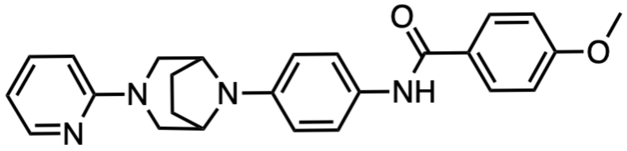

Fig. S1. Chemical structure of HY-2-15.

Machine Learning Datasets

A total of 315 binding measurements were collected. The dataset exhibits reasonable class balance, comprising 138 BF-2846/M503 class compounds, 121 BV-21 class compounds, and 56 TZ61-84 class compounds. A quantitative visualization of the 3D similarity between the complete compound set and each of the three class parents, as well as BF-2846, is presented in Figure S2. 3D Tanimotos were computed according to maximal molecular overlap obtained after running a greedy minimization algorithm. The entire dataset was subjected to the same procedure for all four parent compounds to produce the data in Figure S2.

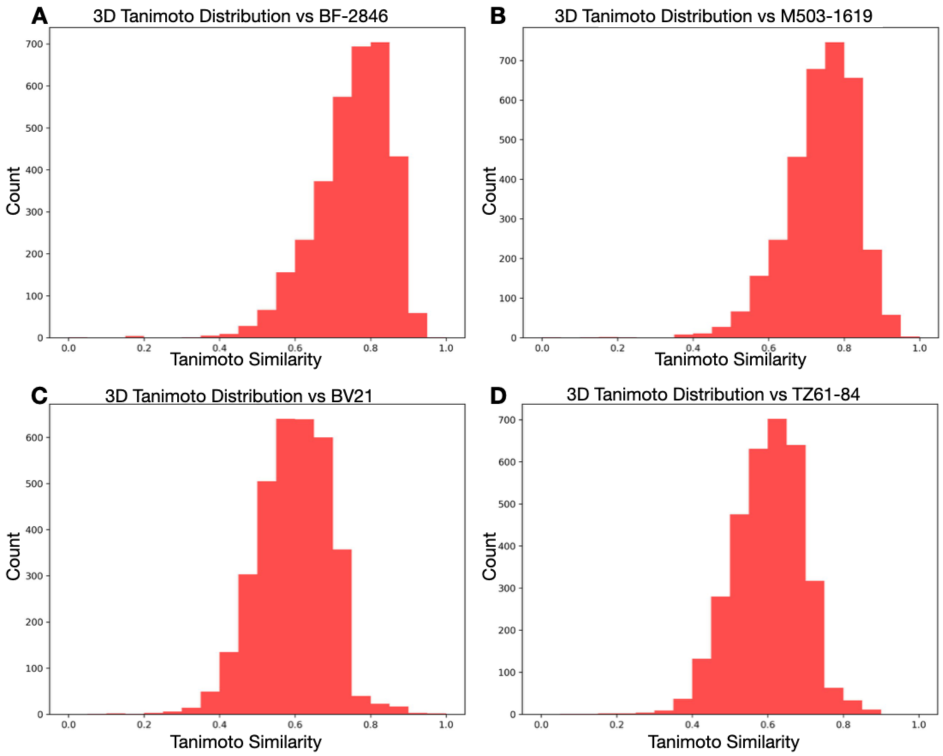

Fig. S2. Data set distribution shown by 3D Tanimoto histograms compared to the structural classes: (A) 3D Tanimoto histogram compared to BF2846; (B) 3D Tanimoto histogram compared to M503; (C) 3D Tanimoto histogram compared to BV21; D) 3D Tanimoto histogram compared to TZ61-84.

We allocated around 85% of the data for model training with five-fold cross-validation and reserved around 15% as an independent test set. To maintain representative class proportions, stratified splitting was applied, resulting in a binder ratio of approximately 20% in both training and test datasets. Table S1 summarizes the

training and test datasets, detailing the number and proportion of binder and non-binder data points in each set. These datasets are available at: https://github.com/ejp-lab/EJPLab_Computational_Projects/tree/master/%CE%B1-SynucleinBinder/Data.

Table S1. Statistics of machine learning sets measured by [^{3}H]BF-2846 displacement assays

Set	Binder Datapoints	Non-binder Datapoints	Binder Ratio	Non-binder Ratio
Train	55	216	0.20	0.80
Test	9	35	0.20	0.80
Total	64	251	N/A	N/A

Model Training

Given the limited training dataset size (271 datapoints), we chose algorithms with inherently higher bias and lower variance: logistic regression, k-nearest neighbors, and decision tree classifier models. Molecules were featurized using Morgan fingerprints and Mordred descriptors, which capture structural and substructural information relevant to molecular similarity and physicochemical properties[2, 3]. Model performance was optimized through hyperparameter tuning and feature selection using five-fold cross-validation, targeting the macro F1 score. Cross-validation was employed rather than allocating a separate validation set to ensure maximal use of the available data for both training and evaluation. The final models were selected from the best trial of Bayesian hyperparameter searching with the TPE sampler in the Optuna Python library[4]. We trained logistic regression, k-nearest neighbors, and decision tree classifier models. The classification reports of the best-performing models during cross-validation are presented in Tables S2–S4 for the logistic regression, k-nearest neighbors, and decision tree classifiers, respectively. To establish an intuitive point of reference, we constructed a straightforward similarity-based baseline

classifier grounded in cheminformatics practices. Specifically, we generated circular Morgan fingerprints (radius = 3, 1024-bit vectors) for every compound in the dataset using RDKit. For each test molecule, we computed its pairwise Tanimoto similarity against all training-set binders only. Each molecule was assigned a binder or non-binder label based on whether its maximum Tanimoto similarity to any training-set binder exceeded a threshold τ. We selected $\tau = 0.5$ because thresholds substantially higher than this approach near-identity similarity and therefore do not provide a meaningful predictive baseline. The classification report for the similarity-based baseline classifier is shown in Table S5.

Table S2. Classification report of the best-performing model during cross-validation of the logistic regression model

	Precision	Recall	F1
Non-binder	0.94	0.76	0.84
Binder	0.47	0.82	0.60
Accuracy			0.77
Macro	0.70	0.79	0.72
Weighted	0.84	0.77	0.79

Table S3. Classification report of the best-performing model during cross-validation of the k-nearest neighbors model

	Precision	Recall	F1
Non-binder	0.87	0.95	0.91
Binder	0.71	0.44	0.54
Accuracy			0.84
Macro	0.79	0.69	0.72
Weighted	0.83	0.84	0.83

Table S4. Classification report of the best-performing model during cross-validation of the decision tree classifier model

	Precision	Recall	F1
Non-binder	0.93	0.77	0.84
Binder	0.47	0.78	0.59
Accuracy			0.77
Macro	0.70	0.77	0.71
Weighted	0.83	0.77	0.79

Table S5. Classification report for the similarity-based baseline classifier

	Precision	Recall	F1
Non-binder	1.00	0.46	0.63
Binder	0.32	1.00	0.49
Accuracy			0.57
Macro	0.66	0.73	0.56
Weighted	0.86	0.57	0.60

Model selection for predicting the prospective set was based on test set performance. The test metrics for the k-nearest neighbors and decision tree classifiers are provided in Tables S6–S7, while those for the logistic regression model are reported in the main text. Among all models, the logistic regression model achieved the best overall performance on test set. A confusion matrix plot for the test set is shown in Fig. S3.

Table S6. Classification report of the hyperparameter-tuned k-nearest neighbors model on test set

	Precision	Recall	F1
Non-binder	0.84	0.91	0.87
Binder	0.50	0.33	0.40
Accuracy			0.79
Macro	0.67	0.62	0.64
Weighted	0.77	0.79	0.77

Table S7. Classification report of the hyperparameter-tuned decision tree classifier model on test set

	Precision	Recall	F1
Non-binder	0.96	0.68	0.79
Binder	0.42	0.89	0.57
Accuracy			0.72
Macro	0.69	0.78	0.68
Weighted	0.85	0.72	0.75

The best parameters of the logistic regression model are shown in Table S8. The optimized model can be directly applied for α-synuclein fibril binding prediction, and its configuration file is available for download at: https://github.com/ejp-lab/EJPLab_Computational_Projects/blob/master/%CE%B1-SynucleinBinder/Model/cv_selected_logistic_regression.joblib. The selected features are available at: https://github.com/ejp-lab/EJPLab_Computational_Projects/blob/master/%CE%B1-SynucleinBinder/Model/cv_selected_features.joblib.

Table S8. Best hyperparameters obtained by Optuna Bayesian optimization

Set	C	Class_weight	max_iter	random_state	solver	tol
Prospective	45.52397795434362,	balanced	1000	42	saga	0.01

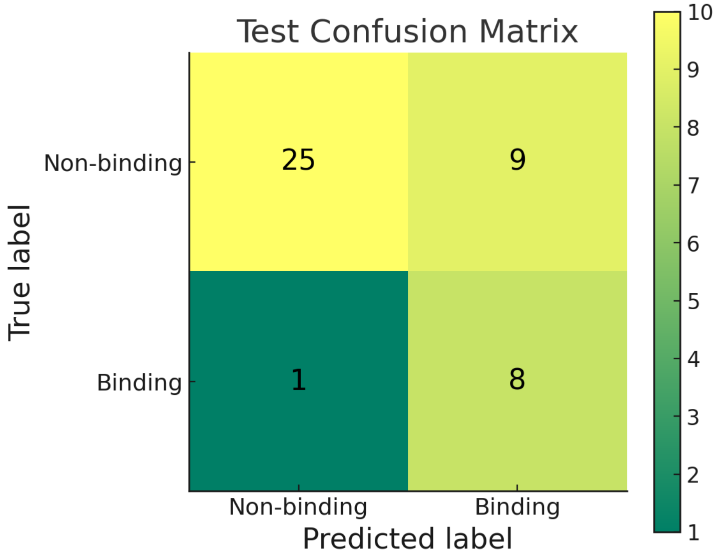

Fig. S3. Confusion matrix plot for test set using optimal logistic regression model

Feature Analysis

To gain insight into which features the classifier relies on, we examined the coefficients of the final logistic regression model trained on selected Morgan fingerprints and Mordred descriptors. For each feature, we extracted the corresponding model coefficient and computed its absolute value as a measure of feature importance. The sign of the coefficient indicates whether the presence of that feature increases (positive sign) or decreases (negative sign) the log-odds of a compound being classified as an α-synuclein fibril binder.

The distribution of the top 40 logistic regression coefficients (Fig. S4) provides a clear view of the molecular features that most strongly influence the model's predictions. The model relies primarily on Morgan (ECFP) bits that encode local atom environments. In addition, several Mordred descriptors also appear among the highest-ranked features, including ATSC, AATS, and GATS autocorrelation descriptors (capturing how atomic properties are distributed across the molecular graph), bonding and unsaturation descriptors such as C1SP2, nBondsM, and nBondsKD, and graph-connectivity indices such as VAdjMat

(explanations of these descriptors are given in Table S9). For completeness, the full list of selected features and their corresponding coefficients is provided in our GitHub repository (https://github.com/ejp-lab/EJPLab_Computational_Projects/tree/master/%CE%B1-SynucleinBinder/Model), enabling readers to examine the learned structure–activity relationships in detail.

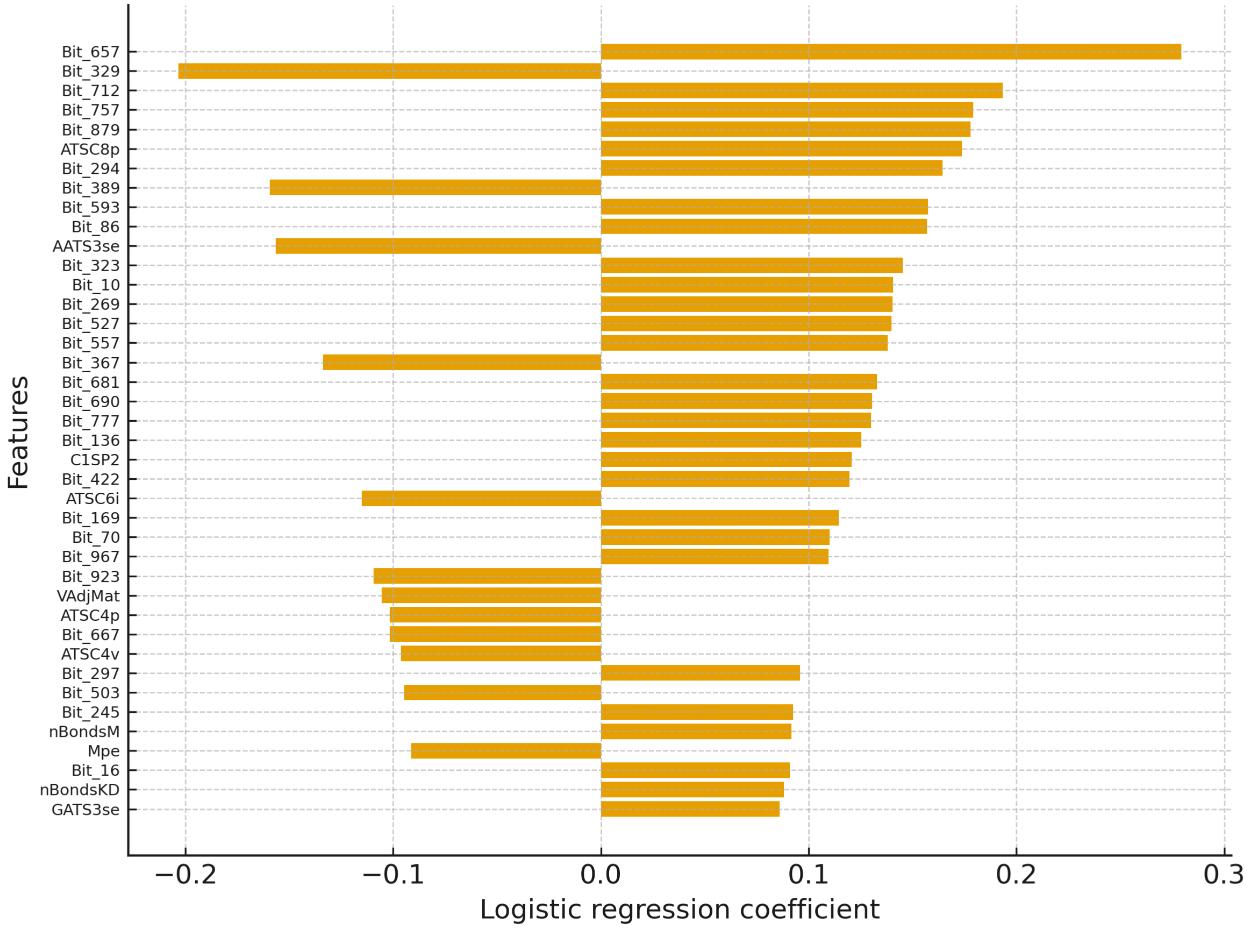

Fig. S4. Top 40 features ranked by logistic regression coefficients

Table S9. Mordred descriptors for key features in logistic regression model

Descriptor	Explanation
ATSC8p	Centered Broto–Moreau autocorrelation at lag 8 weighted by polarizability; measures how atomic polarizabilities correlate across bonds 8 steps apart.
AATS3se	*Average* Moreau–Broto autocorrelation at lag 3 weighted by Sanderson electronegativity; shows electronegativity correlation over 3-bond distances.
C1SP2	Count of sp^2 carbon atoms with exactly one substituent (terminal sp^2 carbons).
ATSC6i	Centered autocorrelation at lag 6 weighted by ionization potential; reflects how ionization energies vary across atoms 6 bonds apart.
VAdjMat	Topological descriptor derived from the adjacency matrix; measures structural branching/complexity based on eigenvalues or matrix variance.
ATSC4p	Centered autocorrelation at lag 4 weighted by polarizability.
ATSC4v	Centered autocorrelation at lag 4 weighted by van der Waals volume (VdW volume correlation at 4-bond separation).
nBondsM	Number of multiple bonds (double, triple, aromatic).
Mpe	Mean atomic polarizability of the molecule.
nBondsKD	Number of Kier–Hall delocalized bonds, indicating resonance/delocalization.
GATS3se	Geary autocorrelation at lag 3 weighted by Sanderson electronegativity; reflects electronegativity variation across 3-bond separations.

Analysis of Training/Test Data Set Diversity

To illustrate the diversity within our 315 compound test and training data sets, in Fig. S5, we show each of the three parent compounds (M503, BV-21, and TZ61-84) with examples of compounds with high (>0.5) and low (0.5-0.2) 2D Tanimoto similarity scores relative to their assigned parent compound class, and low scores relative to the other two compound classes. It also shows an example of an M503/BV-21 hybrid and a BV-21/TZ61-84 hybrid (compounds with 2D Tanimoto scores >0.2 in two categories and less than 0.2 in the other). Finally, Fig. S4 shows examples of alternative scaffold compounds (2D Tanimoto scores <0.2 in all categories). Of the 315 compounds in the test and training data sets, 99 are considered alternative compounds by this criterion, a quantitative measure of diversity in the library.

This figure also provides a visual representation of the degree of similarity represented by certain ranges of 2D Tanimoto scores to allow for an intuitive understanding of the quantitative data. For example,

a single atom substitution of Cl for I in BV-21 accounts for a Tanimoto score of 0.759. Even the low similarity compounds might be viewed as recognizably similar to their parent compounds. However, those compounds classified as alternative scaffolds are significantly different while still sharing general features such as multiple aromatic rings and ≥5 heteroatoms. We note that the range of scores in the 3D Tanimoto analysis of these compounds (Fig. S2) is more compressed. This may be due in part to similar placement of functional groups in 3D conformers in the molecules that is not accounted for in 2D analysis, but caution should be used in comparing the two analyses, as the range of 3D Tanimoto scores is inherently different.

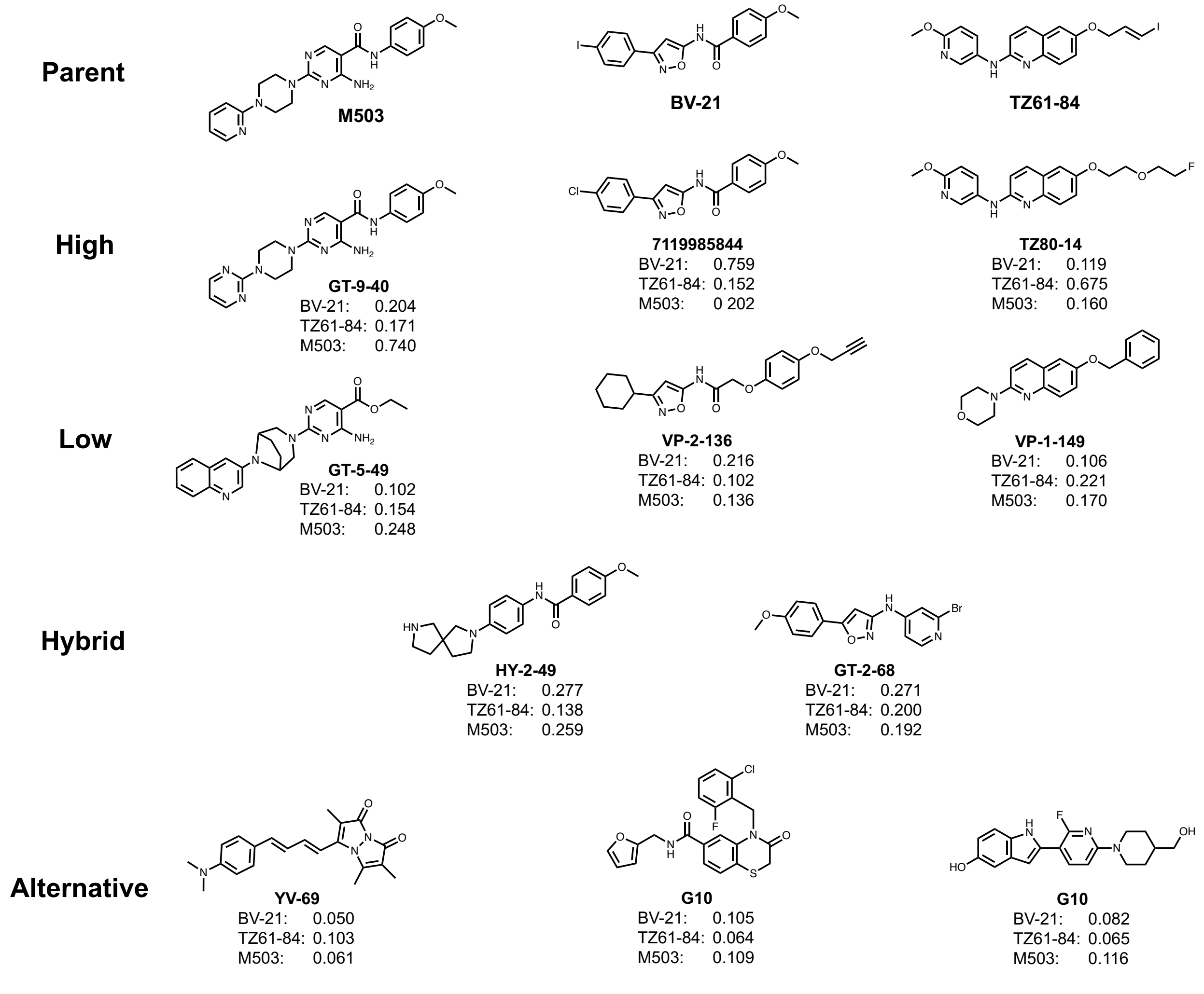

Fig. S5. Illustration of 2D Tanimoto scores for select train/test set compounds. Parent compounds are shown with examples of compounds with High (>0.5) and Low (0.5-0.2) 2D Tanimoto similarity scores relative to their assigned parent compound class, as well as Hybrid compounds (2D Tanimoto scores >0.2 in two categories) and Alternative scaffold compounds (2D Tanimoto scores <0.2 in all categories). Beneath each structure is shown the compound name and its 2D Tanimoto scores relative to the three class parents.

Binder Detection from Mcule library

We curated a prospective dataset from the full Mcule library (downloaded August 04, 2025), comprising 139,655,928 compounds, using a scaffold-aware workflow[5]. The library was obtained from the Mcule website(https://mcule.com/database/). Prior structure-activity relationship (SAR) analysis has focused on three chemotypes for α-synuclein binders, exemplified by **BV-21**, **M503**/**BF-2846**, and **TZ61-84**[1, 6-9]. We assigned each library molecule to its nearest reference scaffold using the Tanimoto similarity coefficient, thereby quantifying structural relatedness between Mcule compounds and predefined parental chemotypes. Each molecule was mapped to the scaffold for which it exhibited the highest Tanimoto similarity. This scaffold-based mapping provided a structural landscape of the library in relation to known chemotypes, highlighting both conserved and novel regions of chemical space. For prospective compound set selection, we deliberately selected a small number of compounds that exhibited high Tanimoto similarity to the reference scaffolds to ensure chemical continuity with validated cores, while also sampling compounds across lower similarity ranges to introduce structural diversity. This balanced, scaffold-aware yet diversity-oriented selection strategy enabled the design of a 30-compound prospective set that both preserved known structural motifs and explored less-charted regions of the chemical space. Note: Due to lack of TZ61-84-type compound availability, three novel compounds were included from in-house libraries. Some of the compounds from Mcule libraries were also available through synthesis.

After selecting the prospective dataset, the final model was applied to predict potential binders within it.

Experimental confirmation of these predictions was obtained via [^{3}H]BF-2846 displacement assays (described above), with summary statistics reported in Table S10. The complete prospective set is publicly available at https://github.com/ejp-lab/EJPLab_Computational_Projects/tree/master/%CE%B1-SynucleinBinder/Data. Model classification metrics on this dataset are provided in Table S11.

Table S10. Statistics of the prospective set obtained from Mcule library and measured by [^{3}H]BF-2846 displacement assays

Set	Binder Datapoints	Non-binder Datapoints	Binder Ratio	Non-binder Ratio
Prospective	9	21	0.3	0.7

Table S11. Classification report of the hyperparameter-tuned logistic regression model on the prospective dataset

	Precision	Recall	F1
Non-binder	0.88	0.71	0.79
Binder	0.54	0.78	0.64
Accuracy			0.73
Macro	0.71	0.75	0.71
Weighted	0.78	0.73	0.74

Examples of binding data for four compounds are shown in Fig. S6 with the fitted curves used in determining K_i. Note that differences in percent displacement of [^{3}H]-BF2846 do not affect the K_i values. Table S12 presents all of the compounds in the prospective set, including their chemical structures, SMILES strings, Mcule IDs, predicted binding classes, K_i values, experimental binding classes, and Tanimoto scores relative to BV-21, TZ61-84, and M503. They are sorted according to the highest Tanimoto score in any class. Compounds below a Tanimoto score of 0.2 for any parent are determined to be alternative scaffolds. This table is also provided as a csv file.

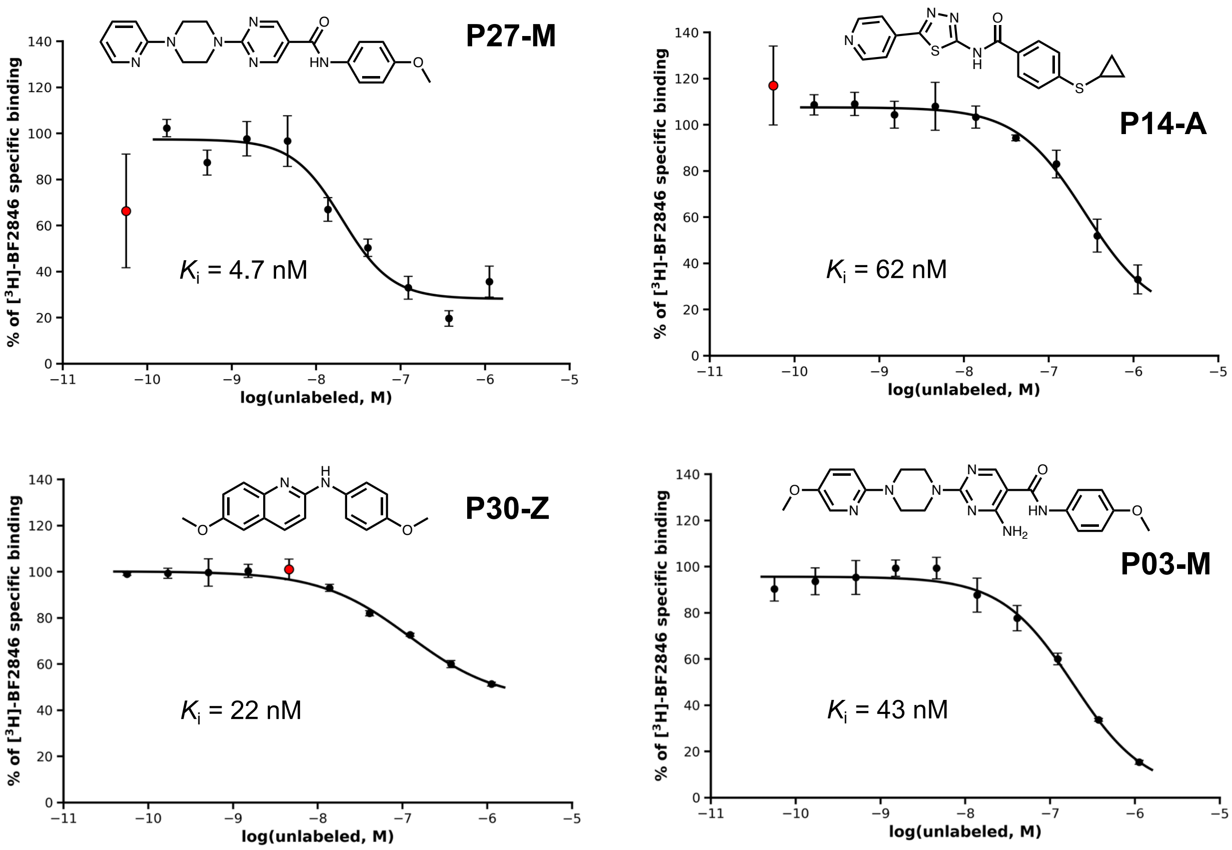

Fig. S6. Example binding data in [^{3}H]-BF2846 displacement assay. Curves were fit as described in Data Fitting section to obtain K_i. Red data points indicate outliers identified in fitting procedure.

Table S12. Prospective compound set prediction, testing, and structural analysis data

Name	SMILES	Mcule ID	Pred.	Ki (nM)	Exp.	FP BV-21	FP TZ61-84	FP M503
**P07-A**	CC=1C=C(N2CC(O)CC2C=3C=CC(F)=CC3)N4N=CN=C4N1	MCULE-8208018033	0	NB	0	0.073	0.103	0.118
**P11-A**	C1CCN(C2=NC=C(C3N=C(C4CN5C(=CN=C5)CC4)ON=3)C=C2)CC1	MCULE-4759842239	0	NB	0	0.096	0.070	0.127
**P06-A**	CCC1=CC=C(C=C1)NC(NC2=CNC3=CC=CC=C23)=O	MCULE-2241433376	0	NB	0	0.102	0.101	0.127
**P10-A**	C1(=NN=C(SCC(=O)NC2=CC=C3C(OCO3)=C2)N1C)C1C=COC=1C	MCULE-6909752187	0	NB	0	0.125	0.095	0.136
**P17-A**	ClC=1C=CN2N=C(NC(=O)NC=3C=NN(C3)C4CCCCC4)N=C2C1	MCULE-8621314498	0	NB	0	0.136	0.105	0.128
**P25-A**	CC1=CC=C(C2NC(SCC(NC3=CC4=C(C=CN4C)C=C3)=O)=NN=2)C=C1	MCULE-6496962235	1	NB	0	0.127	0.115	0.138
**P02-A**	NC1=NC(C2=CC=C(N(C)C)C=C2)=CS1	MCULE-3640234203	0	NB	0	0.143	0.087	0.106
**P09-A**	CC(CN(CC1)C(Nc(cc2)ccc2C(C)=O)=O)N1c1ccc(C)cc1	MCULE-8493756119	0	NB	0	0.134	0.089	0.145
**P16-A**	CC(C)N1C=C(NC(=O)NC=2C=CC3=C(OCC3(C)C)C2)C=N1	MCULE-9820771788	0	NB	0	0.147	0.112	0.137
**P15-A**	CC(C)C1=NC=2C=C(NC(=O)NC=3C=C(C=CN3)C(=O)N(C)C)C=CC2O1	MCULE-8008821641	0	NB	0	0.148	0.124	0.138
**P26-A**	CC(C1OC(C2=CC=CC=C2)=CN=1)NC(C(NC1=CC2=C(N=C(O2)C)C=C1)=O)=O	MCULE-5206636263	0	NB	0	0.143	0.148	0.171
**P13-A**	CC(C)NC=1C=CC(=CN1)C(=O)NC2=NN=C(S2)C=3C=CN=CC3	MCULE-5266123310	1	104	0	0.179	0.117	0.153
**P14-A**	O=C(NC1=NN=C(S1)C=2C=CN=CC2)C=3C=CC(SC4CC4)=CC3	MCULE-6206705061	0	61.74	0	0.188	0.077	0.111
**P22-A**	CCN(CC)C=1C=CC(=CC1)C(=O)NC2=NN=C(S2)C=3C=CN=CC3	MCULE-9236698514	1	4.605	1	0.191	0.097	0.113
**P29-A**	CC1=NNC(=N1)C=2C=CC(NC(=O)C3=CC=4C=NC=CC4O3)=CC2	MCULE-2019341366	1	9.414	1	0.196	0.123	0.189
**P12-A**	[O-][N+](=O)C=1C=CC(=CC1)C(=O)NC2=NN=C(S2)C=3C=CN=CC3	MCULE-7388529879	0	617	0	0.198	0.071	0.096
**P19-V**	O=C(C1=CC=C(C)C(C)=C1)NC(S2)=NN=C2C3=CC=NC=C3	MCULE-4854937769	0	12.7	1	0.202	0.092	0.108
**P23-V**	CCOc(cc1)ccc1C(Nc1nnc(-c2ccncc2)s1)=O	MCULE-1488042594	1	10.36	1	0.244	0.127	0.164
**P24-M**	COC1=CC=C(N=C(N2CCN(C3=NC=CC=N3)CC2)S4)C4=C1	MCULE-1195918550	1	30.524	0	0.158	0.140	0.273
**P08-M**	Cc1nc(N(CC2)CCN2c2ncccc2)nc(CCC2)c1C2=O	MCULE-5461172863	1	NB	0	0.075	0.067	0.323
**P18-Z**	CN(C)C1=CC2=CC=CC=C2N=C1NC3=CN=C(OC)C=C3	TZ80-142	0	NB	0	0.112	0.330	0.157
**P04-V**	O=C(NC1=NN=C(C2=CC=NC=C2)S1)C3=CC=C(OC)C=C3	MCULE-4152335408	1	47.9	0	0.350	0.143	0.192
**P05-Z**	CN(C)C(C=C1)=CC2=C1N=C(S2)NC3=CN=C(OC)C=C3	TZ80-84	0	257.6	0	0.121	0.352	0.155
**P01-V**	NC1=CC(C2=CC=C(OC)C=C2)=NO1	MCULE-5879131729	0	NB	0	0.375	0.136	0.169
**P30-Z**	COC1=CC=C(N=C(NC2=CC=C(OC)C=C2)C=C3)C3=C1	TZ90-8	1	22.12	1	0.181	0.447	0.200
**P28-V**	BrC1=CC=C(C2=NOC(NC(C3=CC=CC(OCC)=C3)=O)=C2)C=C1	MCULE-1131491708	1	9.87	1	0.457	0.140	0.165
**P20-V**	CC1=CC=C(C2=NOC(NC(C3=CC=C(OCCC)C=C3)=O)=C2)C=C1	MCULE-6705203185	1	4.25	1	0.583	0.145	0.140
**P27-M**	O=C(C1=CN=C(N=C1)N2CCN(CC2)C3=NC=CC=C3)NC4=CC=C(OC)C=C4	MCULE-1682064949	1	6.77	1	0.227	0.159	0.646
**P03-M**	O=C(C(C=NC(N1CCN(CC1)C2=CC=C(OC)C=C2)=N3)=C3N)NC4=CC=C(OC)C=C4	MCULE-2502940094	1	44.78	0	0.221	0.185	0.692
**P21-V**	BrC1=CC=C(C2=NOC(NC(C3=CC=C(OC)C=C3)=O)=C2)C=C1	MCULE-5698809192	0	16.62	1	0.783	0.155	0.194

References

1. B. Janssen, G. Tian, Z. Lengyel-Zhand, C. J. Hsieh, M. G. Lougee, A. Riad, K. Xu, C. Hou, C. C. Weng, B. J. Lopresti, H. J. Kim, V. V. Pagar, J. J. Ferrie, B. A. Garcia, C. A. Mathis, K. Luk, E. J. Petersson and R. H. Mach, *Mol Imaging Biol*, 2023, **25**, 704-719.
2. H. L. Morgan, *Journal of Chemical Documentation*, 1965, **5**, 107-113.
3. H. Moriwaki, Y.-S. Tian, N. Kawashita and T. Takagi, *Journal of Cheminformatics*, 2018, **10**, 4.
4. T. Akiba, S. Sano, T. Yanase, T. Ohta and M. Koyama, 2019.
5. R. Kiss, M. Sandor and F. A. Szalai, *Journal of Cheminformatics*, 2012, **4**, P17.
6. H. Zhao, T. Huang, D. D. Dhavale, J. Y. O'Shea, Z. Lengyel-Zhand, D. S. Guarino, J. Gu, X. Yue, Y.-H. Nai, H. Jiang, M. G. Lougee, V. V. Pagar, H. J. Kim, B. A. Garcia, E. J. Petersson, C. A. Mathis, P. T. Kotzbauer, J. S. Perlmutter, R. H. Mach and Z. Tu, *Journal*, 2025, **14**.
7. J. J. Ferrie, Z. Lengyel-Zhand, B. Janssen, M. G. Lougee, S. Giannakoulias, C. J. Hsieh, V. V. Pagar, C. C. Weng, H. Xu, T. J. A. Graham, V. M. Lee, R. H. Mach and E. J. Petersson, *Chem Sci*, 2020, **11**, 12746-12754.
8. G.-L. Tian, C.-J. Hsieh, D. S. Guarino, T. J. A. Graham, Z. Lengyel-Zhand, A. Schmitz, W. K. Chia, A. J. Young, J.-G. Crosby and K. Plakas, *RSC Medicinal Chemistry*, 2025, **16**, 2743-2753.
9. H. A.-O. Kim, W. K. Chia, C. A.-O. Hsieh, D. Saturnino Guarino, T. A.-O. Graham, Z. Lengyel-Zhand, M. Schneider, C. Tomita, M. G. Lougee, H. J. Kim, V. V. Pagar, H. Lee, C. A.-O. Hou, B. A.-O. Garcia, E. J. Petersson, J. O'Shea, P. T. Kotzbauer, C. A. Mathis, V. M. Lee, K. C. Luk and R. A.-O. Mach.
